# Activity changes in neuron-astrocyte networks in culture under the effect of norepinephrine

**DOI:** 10.1371/journal.pone.0203761

**Published:** 2018-10-17

**Authors:** Yasmin Bar El, Sivan Kanner, Ari Barzilai, Yael Hanein

**Affiliations:** 1 School of Physics and Astronomy, Tel-Aviv University, Tel-Aviv, Israel; 2 Department of Neurobiology, Faculty of Life Sciences, Tel-Aviv University, Tel-Aviv, Israel; 3 Sagol School of Neuroscience, Tel-Aviv University, Tel-Aviv, Israel; 4 School of Electrical Engineering, Tel-Aviv University, Tel-Aviv, Israel; Aix-Marseille Universite, FRANCE

## Abstract

The concerted activity of neuron-glia networks is responsible for the fascinating dynamics of brain functions. Although these networks have been extensively investigated using a variety of experimental (*in vivo* and *in vitro*) and theoretical models, the manner by which neuron-glia networks interact is not fully understood. In particular, how neuromodulators influence network-level signaling between neurons and astrocytes was poorly addressed. In this work, we investigated global effects of the neuromodulator norepinephrine (NE) on neuron-astrocyte network communication in co-cultures of neurons and astrocytes and in isolated astrocyte networks. Electrical stimulation was used to activate the neuron-astrocyte glutamate-mediated pathway. Our results showed dramatic changes in network activity under applied global perturbations. Under neuromodulation, there was a marked rise in calcium signaling in astrocytes, neuronal spontaneous activity was reduced, and the communication between neuron-astrocyte networks was perturbed. Moreover, in the presence of NE, we observed two astrocyte behaviors based on their coupling to neurons. There were also morphological changes in astrocytes upon application of NE, suggesting a physical cause underlies the change in signaling. Our results shed light on the role of NE in controlling sleep-wake cycles.

## Introduction

Integrated networks of neurons and glial cells are the basis for information processing and encoding in the brain [1]. The communication between neurons and glial cells at different temporal and spatial scales appears to be a fundamental mechanism that affects activity, information processing, and plasticity [1,2].

At the single synapse scale, the tripartite synapse model describes the interplay between a pre-synaptic neuron, a post synaptic neuron, and an astrocyte [3]. Synaptic transmission may activate astrocytes by elevating the internal calcium concentration. This local signaling can induce signaling to neurons, creating a neuronal-glial-neuronal positive or negative feedback loop to enhance or dampen neuronal activity [4–8]. Astrocytes communicate with synapses through various neurotransmitters [9,10] such as the bidirectional transmitter glutamate [6,11], which elicits astrocyte calcium responses. These responses depend on neuronal firing frequency in a nonlinear way and have a cell-dependent threshold frequency [12]. Synaptic activity can be also affected by global processes such as external chemicals applied to populations of cells.

Recent investigations suggest that global processes, in particular neuromodulation, play important roles in brain network dynamics [13,14] by affecting neuron-astrocyte network communication [15]. Triggered by global changes (e.g., behavior or body temperature), neuromodulation regulates and stimulates brain activity by volume transmission, a process by which chemicals spread from the site of secretion through the brain extracellular fluid to affect a large number of cells [16,17]. As such, neuromodulation provides an important control mechanism [18].

Norepinephrine (NE) is a neuromodulator that affects both neurons and astrocytes through different noradrenergic receptors [19] *in vivo* and *in vitro* [20–22]. NE is released from neuron terminals that originating in the nucleus locus coeruleus, which projects to large areas to influence brain activity in response to behavioral demands [23]. NE can alter global processes such as the sleep-wake cycle and shifts in attention. At the cell level, NE stimulates activation of astrocyte calcium signaling [19] and reduces the rate and amplitude of neuronal activity [24]. NE decreases cortical neuronal activity by limiting neurotransmitter release and by increasing the sensitivity to inhibitory signaling [23]. Specifically, NE inhibits glutamatergic synaptic transmission [25] while promoting gamma aminobutyric acid (GABA) activity [26]. Furthermore, NE can alter astrocyte morphology [27–29]. During sleep, low levels of NE lead to astrocytic shrinkage. This phenomenon is part of the function of the so-called glymphatic system. Decreased astrocyte volume results in a larger extracellular space volume and higher interstitial fluid flux and thus lowers ion concentrations and facilitates waste clearance from the brain during sleep [27–29]. Thus, NE affects the activities of populations of cells in brain networks to control a multitude of different behavioral states [28,30].

To date, neuromodulation has been studied with a focus on either neurons or astrocytes at the single-cell level or on neuronal activity in networks. Studies of neuron-astrocyte interactions have been mostly limited to the single synapse level. How neuromodulators influence network level signaling between neurons and astrocytes was generally overlooked. In this investigation, we aim to uncover the effect of neuromodulation on neuron-astrocyte network communications and explored the role of astrocyte signaling in brain networks.

We used a previously described co-culture of neurons and astrocytes as a model system [31,32]. Cultured networks offer several unique opportunities when attempting to study the interplay between neurons and astrocytes. First, isolated astrocyte networks can be obtained and investigated separately. As neurons respond to electrical stimulation (ES) but astrocytes do not [12,33], it is therefore possible to use ES to evoke specific and well controlled activity. Neurons and astrocytes can be imaged and their electrical or Ca^2+^ signals recorded at single-cell, single-action-potential resolution. Indeed, in a previous investigation we used ES to demonstrate a frequency-dependent communication link between neurons and astrocytes [12].

Here, we used the neuron-astrocyte system to study the neuromodulatory effects of NE on neuron-astrocyte communication. Whereas neuron-glia communication is essentially understood at the molecular level, the effects of neuron-glia communication on network dynamics are still poorly understood. Our analysis revealed the dramatic changes in network activity upon application of NE, demonstrating a functional link between neuron and astrocyte networks while accounting for the different time scales and behaviors of the two networks. We showed that under neuromodulation networks behavior and inter-network communication are altered.

## Materials and methods

### Ethics statement

All animal care and experiments presented in this study were conducted according to the animal research guidelines from Tel Aviv University and were approved by the Tel Aviv University Animal Care Committee.

### Primary cortical neuronal-astrocyte cell culture

Cortical cultures were prepared from post-natal day 0 or 1 mice. Cortices from pups were dissected and placed on ice, chopped with scissors in a papain-based dissociation buffer (2.5 mM CaCl_2_, 0.83 mM EDTA, 137 U papain (Sigma-Aldrich)), 100 μl DNAse (Sigma-Aldrich), 3–5 crystals of L-Cysteine (Sigma-Aldrich), HBSS with 20 mM HEPES (pH 7.4) and placed on a rotating shaker for 15 min at room temperature. After centrifuging, the supernatant was discarded, and the pellet was resuspended in modified essential medium (MEM) without L-glutamine with essential amino acids (Beit Haemek, 06-1025-01-1A), 5% heat-inactivated fetal calf serum (Biological Industries), heat-inactivated 5% horse serum (Beith Haemek, 04-004-1), 2 mM glutamine (Beit Haemek, 03-020-1c), 3 mg/ml glucose, 2% B-27 (Gibco, 17504–044), 0.5% Pen/Strep (100 U/ml penicillin, 100 μg/ml streptomycin; Beit Haemek, 03-031-1B) and triturated seven times. Cells were plated on poly-D-lysine (PDL, Sigma-Aldrich, P7405-5MG) coated micro-electrode arrays (MEAs; 200/30iR-Ti-gr and 500/30iR-Ti-pr; Multichannel Systems) [34] with a cell density of 2000–2500 cells/mm^2^ (~10^6^ cells per dish). Cultures were maintained at 37°C with 5% CO_2_. Growth medium was partially replaced every 3–4 days (MEM-EAGLE (without L-glutamine with essential amino acid), 5 mg/ml glucose, 5% heat-inactivated fetal calf serum, 0.8% GlutaMAX (100X; Gibco, 35050–038), 0.5% Pen/Strep, 2 mM glutamine, 2% B-27). Neuron-astrocyte cultures were recorded after 14–19 days *in vitro* (DIV).

### Isolated astrocyte cell culture

**Fig 1:**
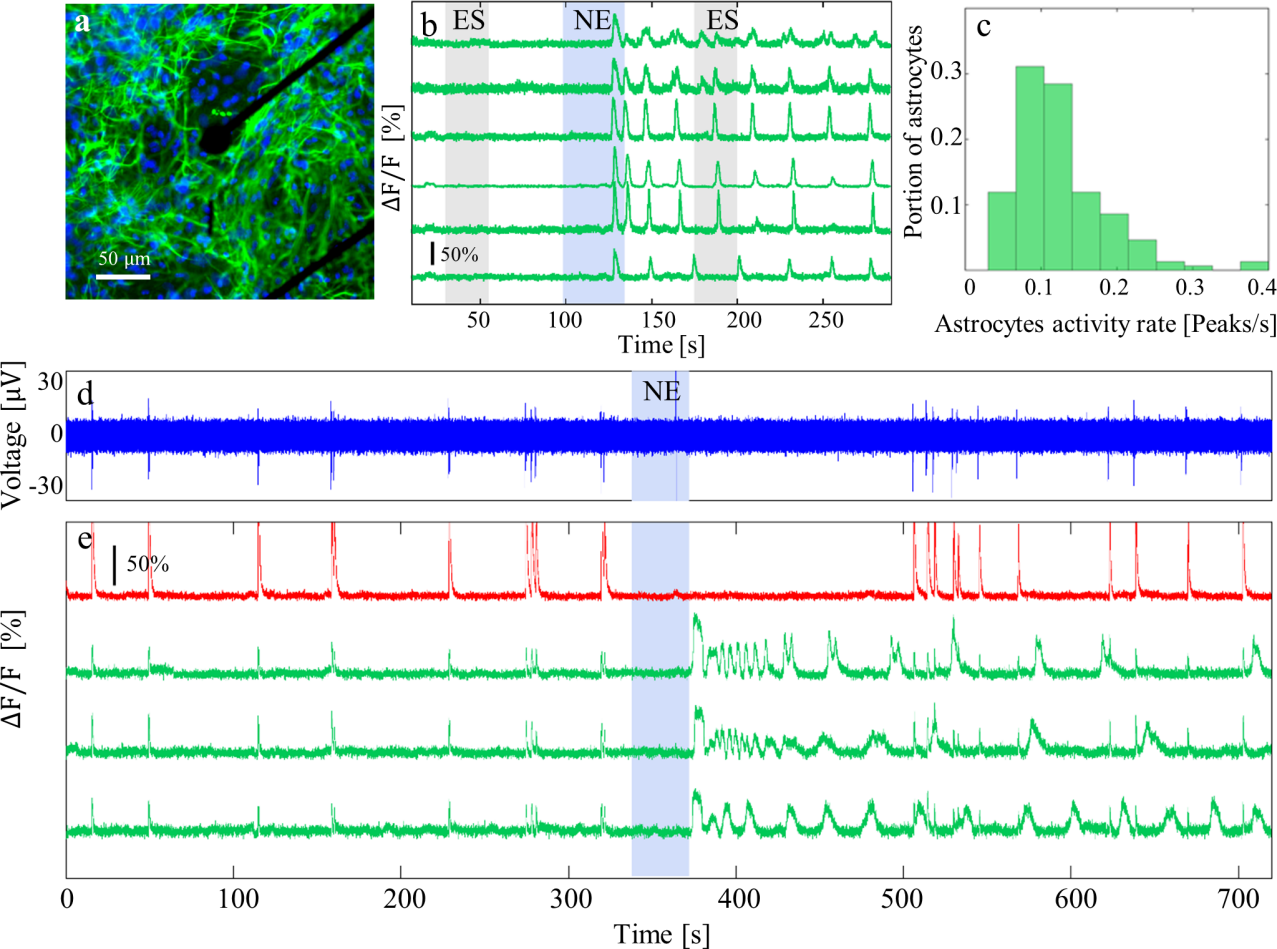
Effects of NE on activities of neurons and astrocytes. (a) Representative image of an isolated astrocyte culture (27 DIV) grown on MEA. Cells were stained with GFAP (green), and nuclei were stained with DAPI (blue). (b) Ca^2+^ traces of six representative astrocytes from an isolated astrocyte culture after ES and NE application. Gray rectangles mark ES periods (Stimulation parameters: 25 s, 2 stimulating electrodes, 25 μA/electrode, 20 Hz). Blue rectangles mark the time of 50 μM NE addition. (c) Astrocytic activity rate (peaks/s) in the first 30 s following NE application, n = 151 cells from 17 different cultures. Bin width, 0.037 peaks/s. (d) Neuronal extracellular voltage (blue) in a mixed neuron-astrocyte culture (16 DIV). (e) Ca^2+^ traces of the same recording as in (d) of a mixed neuron-astrocyte culture, before and after 50 μM NE addition. The trace of a neuron is in red, and traces of three selected astrocytes are in green. Blue rectangle marks the time of NE addition.

Astrocyte cultures were prepared from the primary cortical neuronal-astrocyte cell cultures as detailed above. Two alternative protocols were used for the astrocyte isolation process. In both protocols the growth medium was replaced from a neuronal enriched growth medium to astrocyte enriched growth medium: MEM-EAGLE (without L-glutamine, with essential amino acids) (Beit Haemek), 3 mg/ml glucose, 10% heat-inactivated fetal calf serum (Biological Industries), 0.8% GlutaMAX (100X; Gibco), 0.5% Pen/Strep, 2 mM glutamine (Beit Haemek). Under these conditions, the astrocytes proliferate, but the neurons die after a few days. In the first protocol, the cells were plated on PDL-coated MEAs with the same cell density as used for the neuronal-astrocyte cultures. To isolate the astrocytes, one day after plating, the medium was replaced with astrocyte growth media in order to obtain an enriched astrocyte culture. In the second protocol, the cells were plated in PDL-coated T75 flasks, and the medium was replaced with astrocyte growth media. When the cells reached confluence (after 20 DIV) they were detached using trypsin/EDTA and re-plated on PDL-coated MEAs at the same cell density as used for the neuronal-astrocyte cultures. Electrical and optical activity recordings ensured that there was no neuronal activity. Moreover, an immunostaining test with GFAP was performed to confirm that no neurons were present in the cultures (Fig 1A and S1 Fig). Astrocyte cultures were analyzed after 20–58 DIV (including the proliferation period).

### Pharmacology

Experiments with NE were performed by adding 50 μM NE (Sigma-Aldrich, A0937) to the recording medium. Studies have shown that concentrations in the order of 10 μM elicit calcium responses, oscillations, and intercellular calcium signaling in astrocytes [19,35,36].

### Immunocytochemistry

Samples were washed twice in a phosphate buffered saline (PBS), then fixed with 4% paraformaldehyde (Merck) for 10 min, and left in PBS until staining. For immunocytochemical staining, fixed cultures were washed three times with PBS (10 min each). Next, they were permeabilized with 0.5% triton X-100 (Sigma-Aldrich) in PBS for 10 min. The cultures were blocked with 2% BSA, 10% normal donkey serum, and 0.25% triton X-100 solution in PBS for 1 h at room temperature. The cultures were then incubated overnight with rabbit anti-GFAP (1:400, Sigma-Aldrich, cat# G9269, RRID: AB_477035), mouse anti-NeuN (1:200, Millipore, cat# MAB377, RRID: AB_2298772) at 4°C. The cultures were then washed three times with PBS and incubated for 1 h at room temperature with the appropriate secondary antibodies: Alexa fluor 488 goat anti-rabbit IgG (1:400, Jackson Laboratories) for the detection of GFAP and Cy-3 donkey anti-mouse IgG (1:700, Jackson Laboratories) for NeuN. After washing three times with PBS, the cultures were mounted with aqueous medium containing DAPI (VECTASHIELD Mounting Medium with DAPI, Vector Laboratories, H-1200). Slides were visualized in a Nikon Eclipse ME600 fluorescent microscope equipped with a high-resolution DXM1200C Nikon digital camera or in a Confocal Laser Scanning Platform Leica TCS SP8.

### Electrophysiology and Ca^2+^ imaging

The recording set-up was as described by Herzog et al [31] and Wallach et al [12]. An extracellular voltage recording (EC) of cortical cultures was performed using MEA (60MEA200/30iR-Ti-gr and 60MEA500/30iR-Ti-pr; Multichannel Systems) at a sample rate of 20 KHz, utilizing low noise pre-amplifier board (MEA- 1060-BC, amplifier, gain x1100; Multichannel Systems). Signals from the microelectrodes were collected and stored using MC-Rack data acquisition software (Multichannel Systems). ES was performed using a dedicated four-channel stimulus generator (STG 2004; Multichannel Systems) and a stimulation protocol programed in MC-Stimulus software (Multichannel Systems). Rectangular and biphasic 400 μs-long current pulses of 25–35 μA (1.4–1.7 mC/cm^2^) at frequencies of 2–40 Hz were extracellularly applied by MEA. Stimulation amplitude and frequency were chosen according to a safety map previously established in our lab [12].

Ca^2+^ imaging was performed in an open air environment. The culture medium was replaced by ACSF medium containing 10 mM HEPES (pH 7.4), 4 mM KCl, 1.5 CaCl_2_, 0.75 mM MgCl_2_, 129 mM NaCl, and 10 mM D-glucose. The cultures were loaded with calcium fluorescent dye Oregon-Green BAPTA-I (Invitrogen, 06807). The cultures were incubated for 35 min in 1 ml ACSF supplemented with 1 μl of 10% pluronic acid F-127 (Biotium59000) and 1 μl Oregon-Green BAPTA-I AM previously diluted with 6.7 μl anhydrous DMSO. During recordings, the cultures were kept at 37°C. Time lapse data were performed at 2x2 binning mode for resolution of 500x502 and 51.948 frames per second, taken with an Olympus upright microscope (BX51WI) fitted with an EMCCD camera (Andor Ixon-885) and a 20x water immersion objective (Olympus, UMPLFLN 20XW NA 0.5). Fluorescent excitation was delivered by a 120-W mercury lamp (EXFO x-cite 120PC) coupled with a dichroic mirror with a filter to match the dye spectrum (Chroma T495LP). Camera control utilized the Andor propriety SOLIS software. The effect of bleaching was very moderate and addressed by using normalization of fluorescence values (ΔF/F_0_).

Synchronization between calcium imaging and extracellular recordings was achieved by feeding the TTL output from the CCD into the MEA acquisition board. The recording and stimulation protocol was programed and controlled using MC-Stimulus software (Multichannel Systems). The program included a trigger for CCD activation and a trigger for voltage recording activation. The microscope field of view was centered and focused on the stimulating electrodes and data acquisition was started just prior to the camera recording, thus allowing accurate collection of camera exposure timings.

Recorded data included raw and filtered extracellular voltage recording of 60 micro-electrodes, time stamps of the electrical recording (sampling frequency 20 KHz), triggers of ES, time stamps of CCD frame acquisitions, and time of manual trigger of the pharmacological application. Data were saved in an mcd file by MC-RACK software (Multichannel Systems).

### Data analysis

Data analysis was performed using custom MATLAB scripts (The MathWorks Inc.) described previously [12,31]. Briefly, Ca^2+^ imaging data was stored in the form of an uncompressed Tif library, where pixel values represent fluorescence intensity. Somata boundaries were semi-automatically delineated on the time-averaged Ca^2+^ image, followed by fine manual adjustments. Raw calcium traces were extracted for every cell by averaging over all pixels within the cell boundary. Raw cell traces typically exhibit a decaying baseline characteristic of bleaching effects. This artifact was corrected by computing each time point to be F-F_0_/F_0_, where F is the raw fluorescence intensity and F_0_ is the local baseline fluorescence (i.e., the minimum of the raw fluorescence in the surrounding 20-s time-window). Since analyzed traces are computed as relative to a local minimum, they are strictly positive. Neuronal signals were distinguished from astrocytic signals based on typical dynamic time scales and physiological properties of their calcium signals [37]. The neurons were characterized by fast variations in Ca^2+^ activity during spike onset, whereas the astrocytes exhibited slowly varying signals.

Astrocytes were considered responsive to ES or NE if their traces presented an onset, crossing a threshold of mean plus 2 standard deviations (SD), within 10 s following stimulation and if there was no apparent activity in the 30 s prior to stimulation. Cultures were considered responsive if there were at least five astrocytes in the field of view and at least two of three of the cells were responsive. Neuronal extra-cellular voltage recording were imported and analyzed in MATLAB using the Neuroshare MATLAB toolbox and Data-Manager (Multichannel Systems).

**Fig 2:**
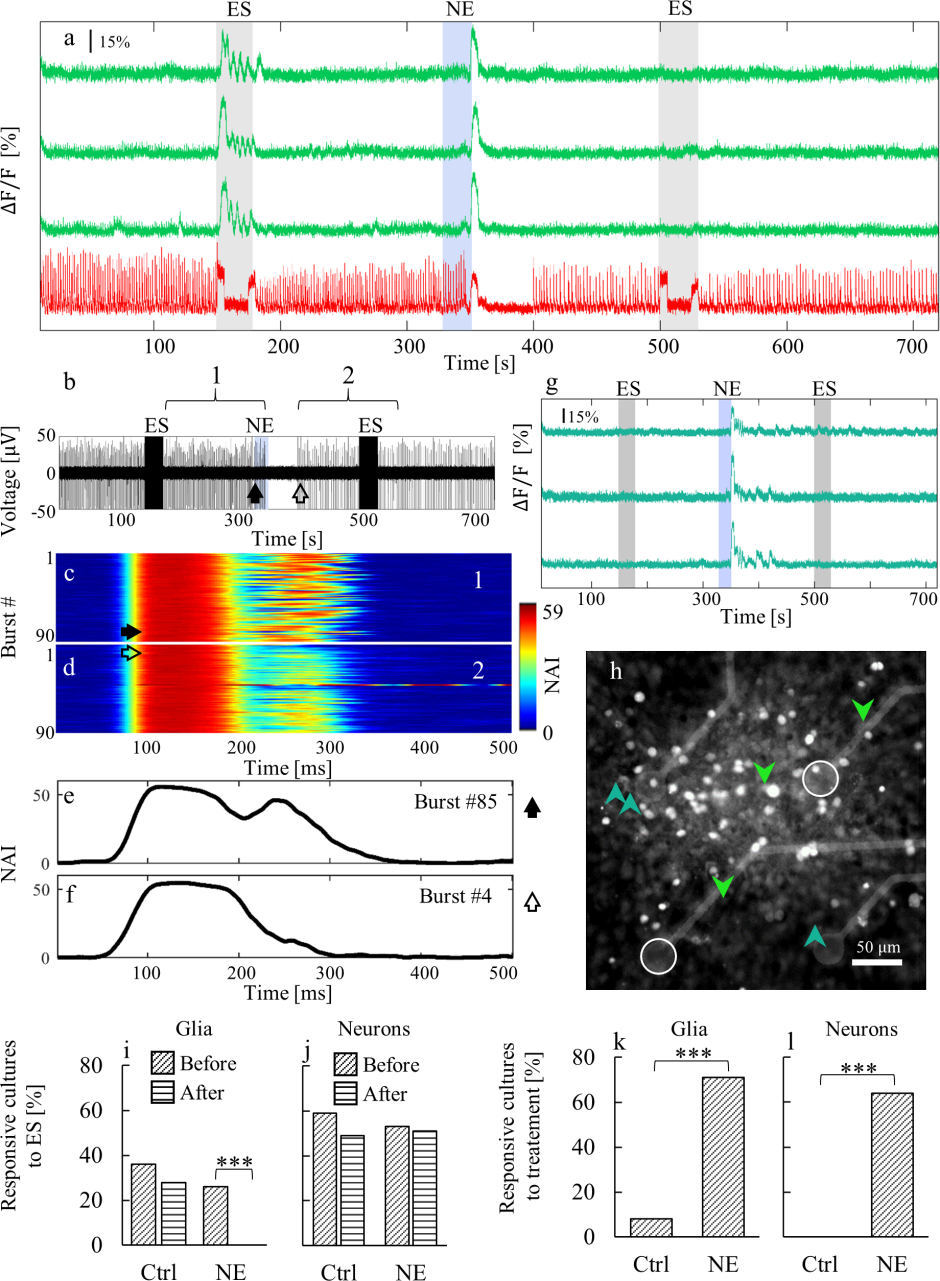
Activities of neuron-astrocyte cultures upon applications of NE and ES. (a) Ca^2+^ traces of three selected astrocytes (green) and a neuron (red) in a mixed culture at 16 DIV. Fluorescence images before and after electrical and chemical stimulations are shown in S4 Fig. (b) A voltage trace of one selected electrode. Segments 1 and 2 refer to the segments presented in (c) and (d) respectively. (c, d) Each panel presents ninety consecutive NAI traces (500 ms each) temporally aligned according to burst onset (c) before and (d) after addition of 50 μM NE. Color-code represents NAI value on a scale of 0 to 59. Numbering 1 and 2 refer to the segments depicted in panel b. Red horizontal line in panel d is an ES artifact, corresponding to the ES period within segment 2 in panel b. (e, f) Representative bursts of NAI before (filled arrow) and after (open arrow) NE addition corresponding to bursts indicated by arrows in panel b. (g) Ca^2+^ traces of three selected astrocytes (turquoise); none were affected by ES. In panels a, b, and g, gray rectangles mark ES periods (30 s, 2 stimulating electrodes, 15 μA/electrode, 10 Hz). Blue rectangles mark the time of NE addition. (h) Representative fluorescence image of the stimulated region of the culture. The astrocytes presented in panel a are indicated by downward-pointing green arrow heads and those in panel g by upward-pointing turquoise arrow heads. The green cells are located around the stimulation electrodes (white circles). The turquoise cells are located further away from the electrodes. (i-l) For comparison, data from 58 cultures from 11 different preparations are shown. (i) Astrocyte responses to ES (control n = 50, NE n = 58). (j) Neuron responses to ES (control n = 37, NE n = 45). (k) Astrocytes activated by NE (control n = 50, NE n = 58). (l) Neurons silenced by NE (control n = 37, NE n = 45). Statistical significance of differences between responsiveness values were measured by Fisher’s test; *** indicates *P* <0.001.

In order to evaluate neuronal network activity, we defined a variable called network activity intensity (NAI) as shown in Fig 2C–2F. Neuronal extracellular voltage recordings by 59 electrodes (a representative voltage trace of one electrode is shown in Fig 2B) were first smoothed by a moving-average filter of length 50 ms. Our aim was to smooth the neuronal bursts to observe changes in the burst envelope before and after treatment. The neuronal burst widths in our cultures ranged from 200 to 1500 ms, i.e., a time window of 50 ms averaged over signals which are smaller than our bursts by a factor of between 0.03 and 0.25 fold. The filter runs over the signal in steps of one sample. In this way, the required envelope is obtained without information loss.

The histogram of the neuronal voltage recordings suggests a bimodal distribution of signal and noise. The minimum between the two Gaussians of signal and noise was automatically calculated for every electrode and was used as a threshold for binarization of the trace. The thresholding was manually verified to ensure accurate detection. Binary traces of the 59 electrodes were then summed to a yield a single trace that represents the NAI with values between 0 and 59 (the number of electrodes crossing the threshold in each time step). The NAI trace was smoothed by a moving-average filter of length 25 ms and down-sampled from 20 KHz to 1 KHz. A threshold of NAI>17 was used to identify network-bursts. After network burst identification, burst time windows of 500 ms (100 ms before threshold crossing and 400 ms thereafter) were extracted. Consecutive bursts (Fig 2C and 2D) were vertically ordered and horizontally aligned according to the time of threshold crossing. The bursts were color-coded according to NAI values.

### Morphology analysis

Cells were seeded in 12-well plates with 500,000 cells per well (~1300 cells/mm^2^). Both test and control (no NE) cultures were treated identically in experiment and in analysis, with the exception of the applied medium, and were analyzed together.

Astrocytic cell morphology analysis was performed using custom MATLAB scripts. Since our cultures are dense and entangled, a separation to individual cells is not applicable. Thus, we developed an algorithm that can be applied globally on the field of view in order to evaluate the fibrousness of the cells in the image without need for segregation into individual cells. The algorithm distinguishes between elongated shapes and spherical shapes and provides information about the fibrousness of the cells by calculation of a score indicative of the total fibrousness of the field of view. The score is calculated as the ratio of boundaries to area (sum over the whole field of view).

The algorithm's steps are as follows: First, a binary image is created by applying a global threshold. The global threshold is calculated using Otsu's method [38] and applied to the original image (S2A Fig). Second, to remove noise effects, an erosion and dilation process is applied to create the binary area image (S2B Fig). The boundaries between the shapes (cell body and processes) and background are obtained applying a four-neighbor gradient on the binary area image. The image of the shape boundaries is then thinned to internal boundaries using a logical AND operation with the binary area image resulting in the boundaries image presented in S2C Fig. Finally, the score is calculated as the ratio between the power (sum over all white pixels) of the boundaries image (S2C Fig) to the power of the total cell area (S2B Fig). A field of view that contains globally cells which are more fibrous will have more boundaries per area and a higher score than a field of view that contains cells which are more spherical and less fibrous. Images of the original field of view, the areas and boundaries, are presented in S2 Fig.

### Statistical analysis

Following a Shapiro-Wilk normality test, data comparisons were carried out using two-tailed Mann–Whitney *U* (MWU) test for two groups. The Fisher test was used to examine the significance of the association (contingency) between the two kinds of classification. All statistical analyses were performed using GraphPad Prism version 6 for Windows. *P*-values <0.05 were considered statistically significant. Significant data are denoted with asterisks: * indicates *P* <0.05, ** indicates *P* <0.01, and *** indicates *P* <0.001. Error bars represent mean ± SEM. Statistical details of individual experiments are given in the figure legends.

## Results

Cultured neuron-astrocyte networks and isolated astrocyte networks were prepared from post-natal day 0 or 1 mice, and experiments were performed after 14 to 58 DIV with or without NE and electrical stimulation (ES). Cell activity was recorded using calcium imaging and multisite extracellular electrical activity using a MEA setup [31]. Cultures showed typical neuronal and astrocytic behavior: Highly synchronized network bursts were observed from neurons that last hundreds of ms in agreement with previously reported studies [39–42]. Astrocytes showed calcium signals of slow onset with slow decay lasting a few s [43].

### NE affects neurons and astrocytes activity

We begin our investigation by exploring the effect of NE on isolated astrocyte and mixed neuron-astrocyte cultures. Isolated astrocytes cultures grown on MEA (Fig 1A) were electrically stimulated and treated with NE (50 μM). Fig 1B shows the effects of ES (gray rectangles) and NE (blue rectangle) on the spontaneous activity of an isolated astrocyte culture. ES did not elicit changes in isolated astrocyte culture activity; it was previously established that astrocytes are electrically non-excitable [33]. Fig 1B shows that upon application of NE, astrocyte networks had a dramatic rise in Ca^2+^ activity. In the astrocyte population of a mixed neuron-astrocyte network, NE had a similar effect (Fig 1E). Inactive cells showed intense calcium signaling activity after NE addition. Astrocytes in isolation and astrocytes in a mixed neuron-astrocyte culture had different patterns of Ca^2+^ activity in response to NE. In astrocyte-only cultures (Fig 1B), NE application caused extracellular calcium wave propagation in the network. In neuron-astrocyte cultures (Fig 1E), astrocyte activity appeared to be oscillatory following NE application. The difference between isolated astrocyte and mixed neuron-astrocyte cultures may be a result of different astrocyte morphologies or network development with and without neurons [44–46].

In the mixed network, a typical astrocyte calcium waveform was observed in response to NE: There was an initial sharp peak followed by small elevations (Fig 1E). This behavior is in agreement with previous investigations on NE as a chemical stimulator of astrocytic calcium activity [19]. Astrocytes in both isolated and neuron-astrocyte mixed cultures had calcium activity at the first 30 s period following NE application at rates spanning the range 0.04 to 0.4 Hz (centered at 0.13 ±0.06 Hz, Fig 1C). The range of astrocytes activity rate was in line with astrocyte rhythms previously reported [11,47]. Neurons responded to NE application with a period (20–150 s) of silenced followed by recovery to spontaneous activity (Figs 1D, 1E, 2A and 2B). The recovered activity had altered burst properties relative to properties prior to NE application, however. To quantify activity, we defined a measure of network activity intensity (NAI) that is calculated from the voltage recordings (see Materials and Methods). After NE application, NAI bursts became shorter, and no oscillation was observed (Fig 2C–2F). There was also a change in neuronal burst frequency: Inter-burst intervals (IBI) became longer (Fig 2A and 2B and S3 Fig) or shorter (Fig 1D and 1E). Thus, the two networks, neuronal and astroglial, had opposite responses to NE application.

### NE affects neuron-astrocyte communication

To evaluate how NE influences neuron-astrocyte glutamate-mediated communication, we examined co-cultured networks under ES. ES is known to affect neuronal activity but does not directly affect astrocytic activity [12,33]. As demonstrated above (Fig 1B), isolated astrocyte cultures did not respond to ES. Fig 2A shows Ca^2+^ traces of a neuron and astrocytes in a mixed neuron-astrocyte culture before, after and during two ES periods (gray rectangles) and one NE application (blue rectangle). Before ES, neurons show spontaneous bursts, whereas astrocytes show sparse and sporadic spontaneous activity. During ES (10 Hz, 30 s), the neurons responded with high frequency and high amplitude activity followed by a decay (saturation effect). A clear response of astrocytic calcium activity during ES was also apparent, parallel in time with the neuronal response (Fig 2A).

This neuronal and astrocyte response has the same manifestation and were obtained by the same set up and protocol as in our previous investigation. These results echo our previous findings of glutamate-mediated, frequency-dependent neuron-astrocyte communication [12]. In the previous study, we showed that the astrocyte response to ES is mediated by glutamate released by nearby neurons. Thus we indicated that the astrocytes are coupled to electrically stimulated neurons and that when the neuronal firing rate crosses a threshold frequency, release of glutamate activates astrocytes. NE application caused a silence period of approximately 50 s in neuronal spontaneous activity and no change in astrocytes activity. The second period of ES, which followed NE application, had the same effect on the neurons as the first ES, but there was no response in astrocytes (Fig 2A). This observation shows that glutamate-mediated neuron-astrocyte signaling is perturbed by NE. Consecutive images of fluorescence, showing the spatial distribution of the culture's calcium response to ES and NE are presented in S4 Fig. These images were collected from the same recording and at the same times as the calcium traces shown in Fig 2A and 2G.

We observed two distinct astrocytic behaviors: astrocytes activated by neurons upon ES (Fig 2A, green traces) and astrocytes not activated by neurons under ES (Fig 2G, turquoise traces). The neuron-coupled astrocytes, which are located close to the stimulating electrodes (Fig 2H, green arrows), responded to ES before NE application and did not respond to NE with an increase in calcium activity as can be seen in the astrocytes calcium traces in Fig 2A. Astrocytes located further away from the stimulating electrodes (Fig 2H, turquoise arrows in) showed no calcium response to ES as can be seen in Fig 2G. These astrocytes that are not coupled to electrically stimulated neurons did respond to NE application with an increase in Ca^2+^ activity, showing the typical calcium elevation waveform (Fig 2G) which was also observed in the mixed culture with no ES periods (Fig 1D). This increase in calcium activity upon NE application was similar to the rise observed in the isolated astrocyte culture upon NE application (Fig 1B) with a different waveform.

### Comparative investigation

The activity of neuron-astrocyte cultured networks is characterized by large inter-network variability [39], and activity characteristics may also vary over time. To establish the generality of our results, the experiments described above were repeated in 58 different cultures (from 11 different preparations). Fig 2I–2L shows comparative results over 58 recorded cultures. In 71% the cultures, astrocytes responded to NE application with a dramatic rise in calcium activity. In control conditions, in which fresh medium was added instead of NE, only 8% of the cultures showed increased calcium signaling in astrocytes. Neurons in 64% of the cultures responded to NE with a period of silence, whereas in control experiments none of the cultures had such a response. The glutamate-mediated communication between the two networks was abolished by the presence of NE in all recorded cultures in which there was a clear communication between neurons and astrocytes before NE application. Astrocytes did not respond to ES after the application of NE, whereas neurons did. Thus, the communication between the two networks was perturbed compared to the control experiment in which there was a response of astrocytes to ES after the addition of fresh medium (S5 Fig).

### NE alters astrocyte morphology

Morphological plasticity is associated with various functions of astrocytes in the brain [48]. As neurons and astrocytes are coupled chemically as well as mechanically, we explored the morphologies of astrocytes in the networks. Astrocyte morphologies were examined under the same conditions used in analyses of Ca^2+^ activity in order to relate morphological changes to changes in signaling. Fourteen DIV neuron-astrocyte cultures were fixed and stained with GFAP after exposure to recording medium (control) or to 50 μM NE for time periods relevant to changes in signaling (1, 5, and 40 min). Individual astrocytes were previously shown to become stellate shaped after periods more than 30 min of exposure to NE [49–51]. Nonetheless, morphological changes in astrocytes can occur in shorter periods of time [52–54].

**Fig 3:**
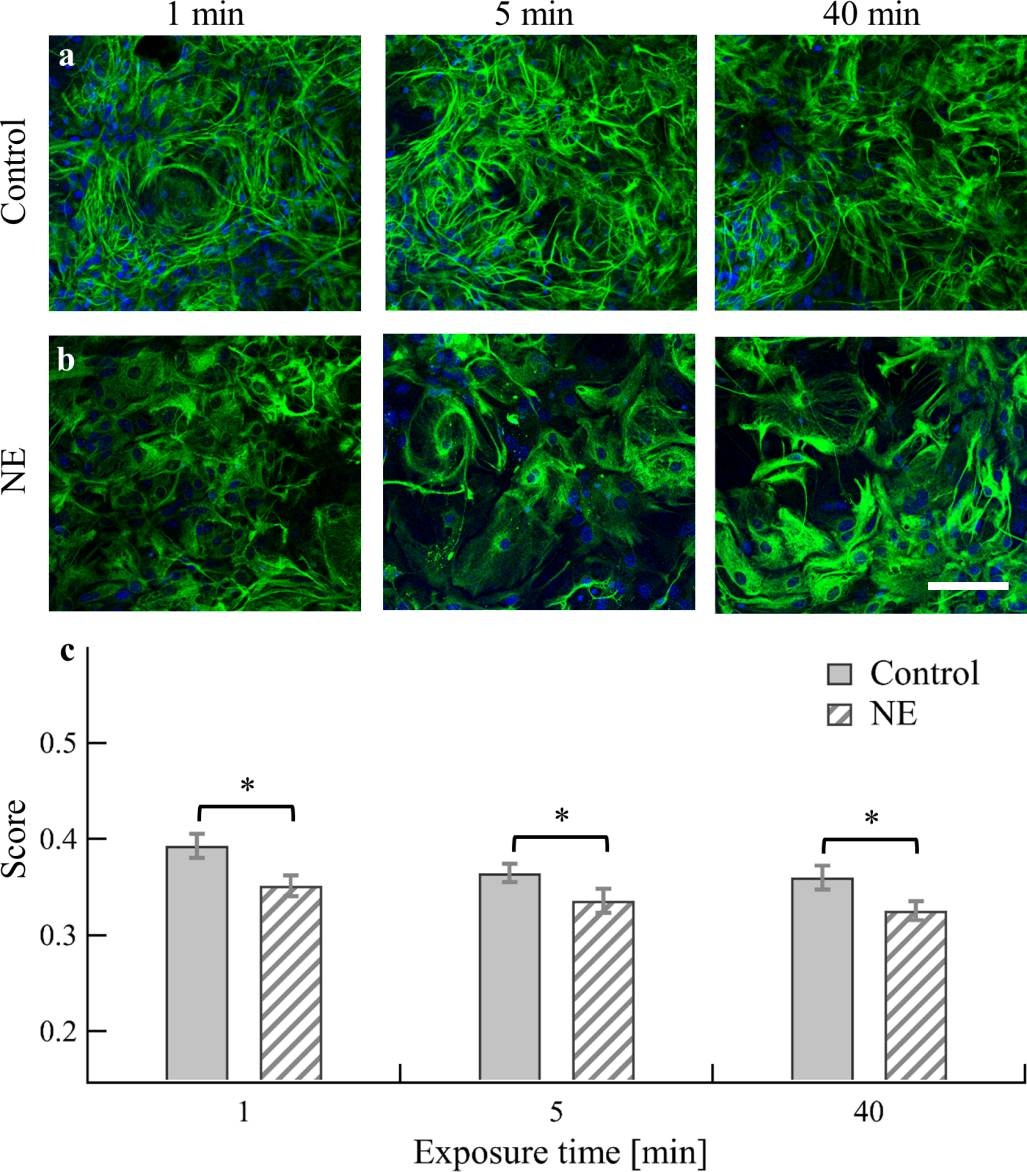
Morphological changes of astrocytes in neuron-astrocyte cultures upon application of NE. (a, b) Representative images of a) control and b) NE-treated mixed cultures at 14 DIV, of 1, 5, and 40 min (left to right) following treatment with medium or 50 μM NE, respectively. Astrocytes were labeled with GFAP (green). Nuclei were stained with DAPI (blue). Scale bars, 100 μm. (c) Comparative analysis of the morphological changes. Bars represent mean score (the ratio of boundaries to area in every field of view). Error bars are SEM (1 min, n = 2 cultures, 18 fields of view; 5 min, n = 3 cultures, 31 fields of view; 40 min, n = 3 cultures, 23 fields of view). Statistical significance of differences between morphological score values were measured using the two-tailed MWU test; * indicates p<0.05. All cultures (control and NE-treated) were seeded at the same seeding density and are presented here in the same size scale.

Here we examined changes in astrocyte morphology associated with the cell network following NE application (or medium for the control cultures) by applying the same experimental protocol and analysis on both control and test cultures as described above. Morphological changes in astrocytes were clearly apparent after treatment with NE (Fig 3): Control cultures treated with medium only were characterized by a highly fibrous and entangled morphology, whereas cultures treated with NE were uniform and spherical and less fibrous. Due to the entangled character of our cultures and the aim to examine morphology within the network, separation of the image into single cells was not feasible. Thus, the apparent changes were quantified by calculating the ratio of boundaries to the surface of the whole field of view (see Materials and Methods). We examined the changes as a function of exposure time; changes were significant compared to the control experiment at all time points (p<0.05).

## Discussion

The computational power of the brain is built on signaling cells–neurons and glia. The discovery of glial cells as a signaling entity in the brain revealed a missing layer in understanding neuronal networks. Although strong evidence suggests that glial cells take part in information processing and plasticity in the brain [1,55,56], more investigation is needed to better understand how these cells communicate with neurons.

This investigation explored the global effects of neuromodulation on network level signaling and communication between neurons and astrocytes, an aspect which was majorly overlooked so far. We used norepinephrine (NE) to modulate neuron and astrocyte network activity and communication *in vitro*. The *in vitro* cultures manifested many features well documented in the literature. We showed that application of NE to cultures of neurons and astrocytes globally affects network dynamics, inter-network communication, and cell morphology. Furthermore, we differentiated between two subgroups of astrocytes based on their coupling to neurons and response to NE.

The molecular mechanisms underlying the effects of NE on neurons and astrocytes at the cellular level and on their signaling have been the subject of intensive investigation of the past 50 years [23,57]. NE is a neuromodulator that affects neuronal and glial cell activity through adrenergic receptors. Neurons and glia cells in culture express both α and β adrenergic receptors and their subunits as in the living brain [20–22]. NE induces calcium activity in astrocytes through α adrenergic receptors 1 and 2 [19,23,58,59]. NE causes the activation of inositol 3 phosphate-mediated mechanism that leads to calcium oscillations between the cytosol and the intracellular calcium stores [60–62]. Our results indeed show a remarkable rise in calcium activity of astrocytes both in isolation and in mixed neuron-astrocytes cultures upon treatment with NE. NE has an inhibitory effect on synchronized neuronal activity as it changes the state of slow-wave sleep and limits paroxysmal depolarization through α2 and β adrenergic receptors [30,63–65]. Our *in vitro* cultures showed spontaneous synchronized neuronal bursts in a variety of patterns and frequencies, in agreement with a previous report [39] before NE application, and marked changes, mainly reduction of the activity, after NE treatment. These results emphasize the inhibitory effect of NE on highly synchronized neuronal activity. Neurons and astrocytes responded to NE application in opposite manners.

In *in vitro* studies NE inhibits neuronal activity by causing hyperpolarization of neuronal cell membranes [66–68] in a process mediated by *β* adrenergic receptors [24,69] and *α*2 adrenergic receptors [70]. This hyperpolarization is generated through signal transduction cascade involving G-proteins and cyclic adenosine-monophosphate (cAMP), leading to an inward rectifying potassium current. NE also increases the frequency of GABA-mediated inhibitory postsynaptic potentials via activation of α1 adrenergic receptors [71] and suppression of excitatory postsynaptic potentials [72]. Moreover, stimulation of α2A adrenergic receptor inhibits excitatory synaptic transmission through a post-synaptic mechanism [73]. As reviewed in [74], activation of α2 and α1 adrenergic receptors temporarily inhibits neuronal activity by decreasing glutamatergic α-amino-3-hydroxy-5-methyl-4-isoxazolepropionic receptor (AMPAR)-mediated transmission and enhancing GABA adrenergic receptor-mediated synaptic responses. Some or all of these known mechanisms may underlie our observations regarding the temporary inhibition of neuronal firing following NE application to the mixed neuron-astrocyte cultures.

Glutamate-mediated neuron-astrocyte communication was demonstrated in our previous investigation [12]. The current research builds on these results to investigate neuron-astrocyte network communication using the same basic experimental set-up and cultures. Specifically, to activate neuronal and astrocyte responsiveness to ES, we used the stimulation properties as scaled in our previous investigation. Here we showed that isolated astrocyte cultures do not respond to electrical stimulation (ES), but in a mixed culture neurons were activated and astrocytes responded if they were coupled to stimulated neurons which firing frequency crossed the threshold to activate the astrocyte calcium response in agreement with our previous study [12]. Furthermore, neuronal and astrocytic calcium signals in response to ES had the same manifestation as was observed in our previous study. Thus, we hypothesize that the same mechanism demonstrated previously of glutamate-mediated neuron-astrocyte communication is at play.

When mixed cultures were treated with NE, this glutamate-mediated neuron-astrocyte communication was abolished. The astrocytes, which prior to NE treatment were coupled to neurons and responded to ES, were not responsive after NE application, even though the neurons responded as they did prior to NE treatment. This indicates that the glutamate-mediated neuron-astrocyte communication was perturbed by NE. It was previously shown that NE influences glutamatergic synaptic outflow [25]. Our observations suggest that glutamate-mediated neuron-astrocyte communication is impaired in the presence of NE because of effects of NE on glutamatergic outflow.

The decrease in synchrony in neuronal activity and the alteration of neuron-glia communication under the influence of NE may be associated with the regulatory effect of NE on sleep-wake cycles [64,65]. The condition of slow-wave sleep is characterized by highly synchronized neuronal activity, which is also observed in our *in vitro* networks. It was previously shown that spontaneous neuronal culture bursts are similar to slow-wave sleep oscillations [75,76]. Thus, cell cultures may serve as a model system for a sleeping network with application of NE causing in a transition to an “awake” state. Experimental data suggest that higher levels of NE alter this synchronization, and the slow-waves phenomenon in sleep that occurs at low NE levels was shown to result from a strong neuron-glia communication circuit initiated by glia [77,78].

We observed two types of astrocyte behavior after NE application based on their communication with neurons. Independent astrocytes, those not in communication with neurons, do not respond to ES but do respond to NE with intensive calcium waves as in experiments in isolated astrocyte cultures or with elevations in calcium signaling as shown in mixed cultures. Neuron-coupled astrocytes respond to ES before NE and do not show calcium activity in response to NE nor to ES after NE application. Following NE application, neuronal activity is silenced for a period of 20–150 seconds and then recovers. After the recovery, neurons respond to ES as they did before NE application. We estimate that this neuronal response to the second ES (the one following NE application) is under the same mechanism as before NE application. Neither independent nor neuron-coupled astrocytes respond to ES when the culture has been treated with NE.

The observation that both astrocytes and neurons are activated during ES in the absence of NE whereas only neurons respond to ES after NE application led us to conclude that loss of neuron-astrocyte communication is due to a change in astrocyte behavior rather than neuronal behavior. This may result from activation of the α1 adrenergic receptors in astrocytes. This activation was shown previously to lead to calcium elevations [23,28]. The elevation of cytosolic calcium ions activates phospholipase, which hydrolyzes membrane lipids, which in turn release arachidonic acid [79]. Arachidonic acid inhibits glutamate uptake by astrocytes [80,81], and results in a lack of astrocyte responsiveness to the glutamate leakage into the synapse during rapid neuronal firing that occurs upon ES. Thus, under NE, astrocytes do not participate in neuron-astrocyte synaptic signaling but intracellular and intercellular calcium signaling do occur.

We also observed morphological changes in astrocytes in the presence of NE. Thus, there appears to be a physical basis for the changes in signaling in the presence of NE. Our analysis was applied on two-dimensional fields of view and cell volume changes could not be evaluated. However, we were able to detect marked changes in cell morphology in short time periods after NE application. Previous studies showed that astrocyte two-dimensional morphology *in vitro* becomes stellate when β-adrenergic receptors are activated by application of NE [49–51]. Furthermore, astrocyte volume *in vivo* increases in the presence of NE [27,29]. In our cultures, we observed significant changes in cell morphology in astrocytes associated with the network upon NE treatment. It is plausible that these morphological changes are associated with the changes in network signaling we observed after NE application.

Previous investigations showed that astrocyte morphology is key to their function and communication with neurons [82]. Specifically, the cell volume and processes morphology can change astrocyte function [83]. This is in agreement with the notion of structure-function relation which is broadly observed in astrocytes variety of functions [82,83].

The well-documented mechanism underlying NE-induced morphological changes in astrocytes is the activation of β adrenergic receptors. This leads to activation of cAMP-dependent protein kinase, which phosphorylates cytoskeletal proteins resulting in morphological changes [29,84,85]. Here we showed that under NE treatment, simultaneous changes in signaling and in morphology of astrocytes occur. Furthermore, the observed morphological change is related to the fibrousness of the cells and therefore the structures of their processes. We thus hypothesize that these two simultaneous changes are related representing structure-function relationship.

The link between neuromodulation and neuron-astrocyte communication is intriguing and may be of relevance to several organ-level functions. Recent studies have shown that during sleep both the interstitial space and the flux of interstitial fluid increase as a result of astrocyte shrinkage. It was suggested that the brain goes through a clearance process (carried out by the glymphatic system) in which potentially neurotoxic waste that accumulates in the central nervous system when animals are awake is removed [27–29]. Dysfunctionality of this process may be at least partially cause the development of brain degenerative diseases such as Alzheimer's disease, which are characterized by the accumulation of protein plaques. Independently, recent studies established the important role of astrocytes in information processing [1,55]. Due to the strong link between activity and morphology of neurons and astrocytes, morphological changes in astrocytes in the neuron-astrocyte network should manifest in changes in astrocytic and neuronal activities.

Overall, we showed robust observations concerning neuron-astrocyte network behavior and communication. Our findings, in particular the link between NE, neurons synchronization and astrocytic activity, are associated with *in vivo* processes and can be further explored with emphasis on disease investigation. Our experiments revealed a clear effect of NE on the coupling between neurons and astrocytes. We observed an independent astrocyte behavior that did not follow neuronal activity, thus shedding light on how astrocytes act as independent signaling entities. This notion suggests new directions to explore as we analyze the role of glia in brain networks.

## Supporting information

S1 Fig Isolated astrocyte culture GFAP immunostaining. Immunostaining images of isolated astrocyte cultures after purification from neurons (prepared as described in the Materials and Methods section titled “Isolated astrocyte cell culture”). Cells were fixed on 27 DIV. Astrocytes were labeled with GFAP (green), neurons with NeuN (red), and nuclei with DAPI (blue). Note, that NeuN-positive cells (neurons) were not detected, showing that astrocyte cultures are composed solely from astrocytes.
(TIF)

S2 Fig Morphological score calculation. A representative image of a fixed neuron-astrocyte co-culture through the image processing steps for morphological analysis score calculation. (a) The original GFAP staining image. (b) The area of cell bodies. (c) Cells boundaries. Scale bar 100 μm. Areas and boundaries were determined by applying the algorithm described in the Materials and Methods section titled “Morphology analysis”. The morphology score of every image (control and test) was calculated as the ratio of the power (sum over all white pixels) of the boundary to the power of the total cell area.
(TIF)

S3 Fig Inter-burst-intervals of neurons before and after treatment. (a) IBI distribution of neurons in control experiment. (b) IBI distribution of neurons in the presence of NE. Bin width for all datasets is 87 ms. (c) Average IBI of control and NE samples before and after treatment. Error bars represent SEM. Statistical significance of differences between IBI distributions were measured using two-tailed MWU test; *** indicates p<0.001. For analysis of calcium imaging data, the fluorescence traces of all identified neurons in the field of view were averaged in order to measure neuronal network activity relying on the highly synchronized character of neuron traces. Bursts and IBIs were measured by applying Hill-Valley analysis on the averaged neuronal trace.
(TIF)

S4 Fig Fluorescence images of mixed neuron-astrocyte culture under perturbations. Temporally ordered selected frames from a movie of calcium imaging recorded under the influence of (a-c) ES and (d-f) NE. (a) Spontaneous activity before the application of ES. The fluorescence is equally spread across the center of the frame. (b) Initiation of ES application on the culture. Electrical current was applied at two microelectrodes (top right and lower left). Stimulation parameters: 2 stimulating electrodes, 25 μA per electrode, 10 Hz. The fluorescence shows two sources of calcium activity, aligned to the stimulating electrode locations. (c) Spreading of the ES onto the network. Fluorescence centers grow wider. (d) Spontaneous activity before the application of NE. The fluorescence is spread across the center of the frame. (e) Calcium image at the time of NE application. The fluorescence intensity grows simultaneously through the whole area, including the margins that did not show fluorescence before. (f) At 10 s after the application of NE. The increased fluorescence is starting to decay yet is still higher than the spontaneous activity baseline. These images were collected from the same recording and at the same times as the calcium traces shown in Fig 2A and 2G. The traces extracted from this recording can be seen in Fig 2A and 2G. Scale bar 100 μm. Culture age 16 DIV.
(TIF)

S5 Fig Neuron-astrocyte spontaneous and electrically evoked activity–control experiment. (a) Extracellular neuronal voltage recording with MEA from a representative electrode. (b) Ca^2+^ traces of selected neurons (red) and astrocytes (green). Periods of ES are marked by gray rectangles. Stimulation parameters: 2 stimulating electrodes, 25 μA/electrode, 10 Hz, 30 s. During the ES, the voltage recording is perturbed. Time of fresh medium addition is marked by the blue rectangle. Culture age 16 DIV.
(TIF)
