# Supplementary figures and images for: Activity changes in neuron-astrocyte networks in culture under the effect of norepinephrine
Source: PLoS One. 2018 Oct 17;13(10):e0203761. doi: 10.1371/journal.pone.0203761 (PMC6192555; doi:10.1371/journal.pone.0203761)

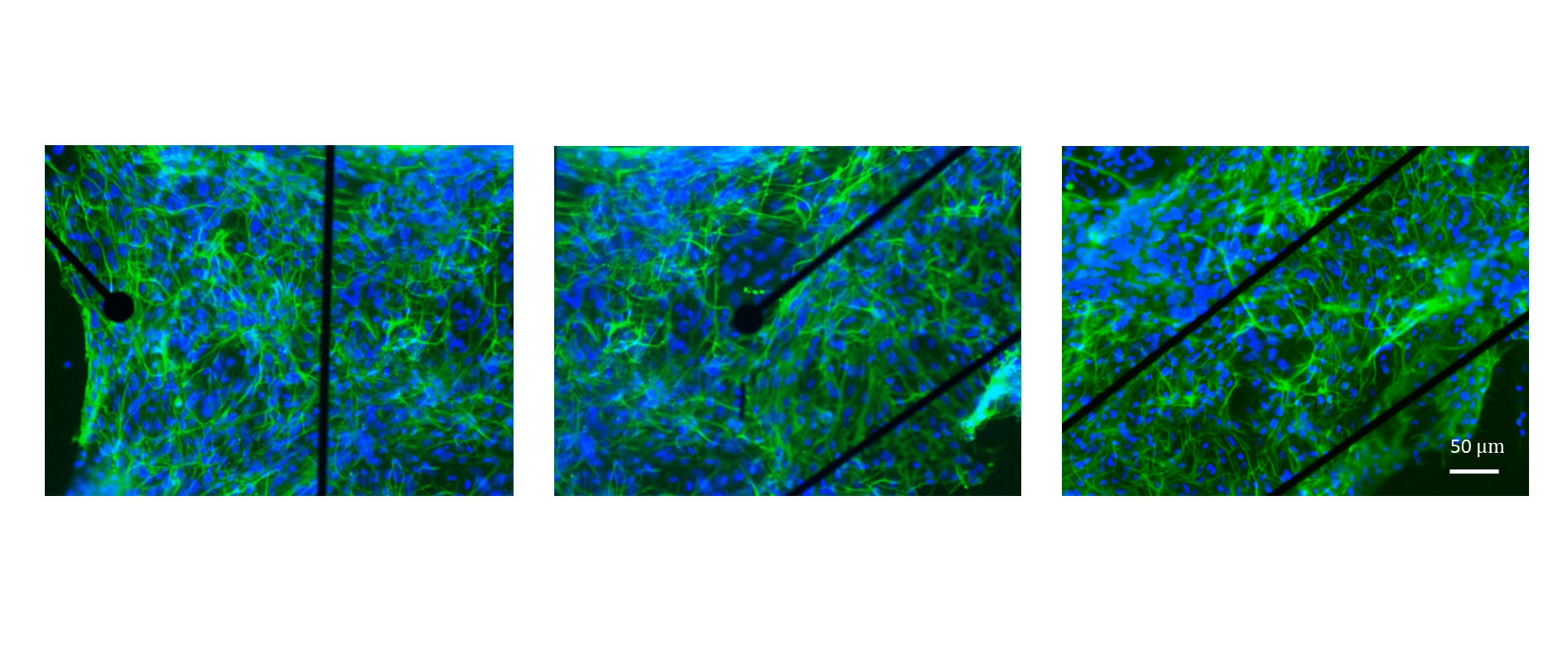

Supplement: S1 Fig — Immunostaining images of isolated astrocyte cultures after purification from neurons (prepared as described in the Materials and Methods section titled “Isolated astrocyte cell culture”). Cells were fixed on 27 DIV. Astrocytes were labeled with GFAP (green), neurons with NeuN (red), and nuclei with DAPI (blue). Note, that NeuN-positive cells (neurons) were not detected, showing that astrocyte cultures are composed solely from astrocytes. (TIF) [file pone.0203761.s001.tif]

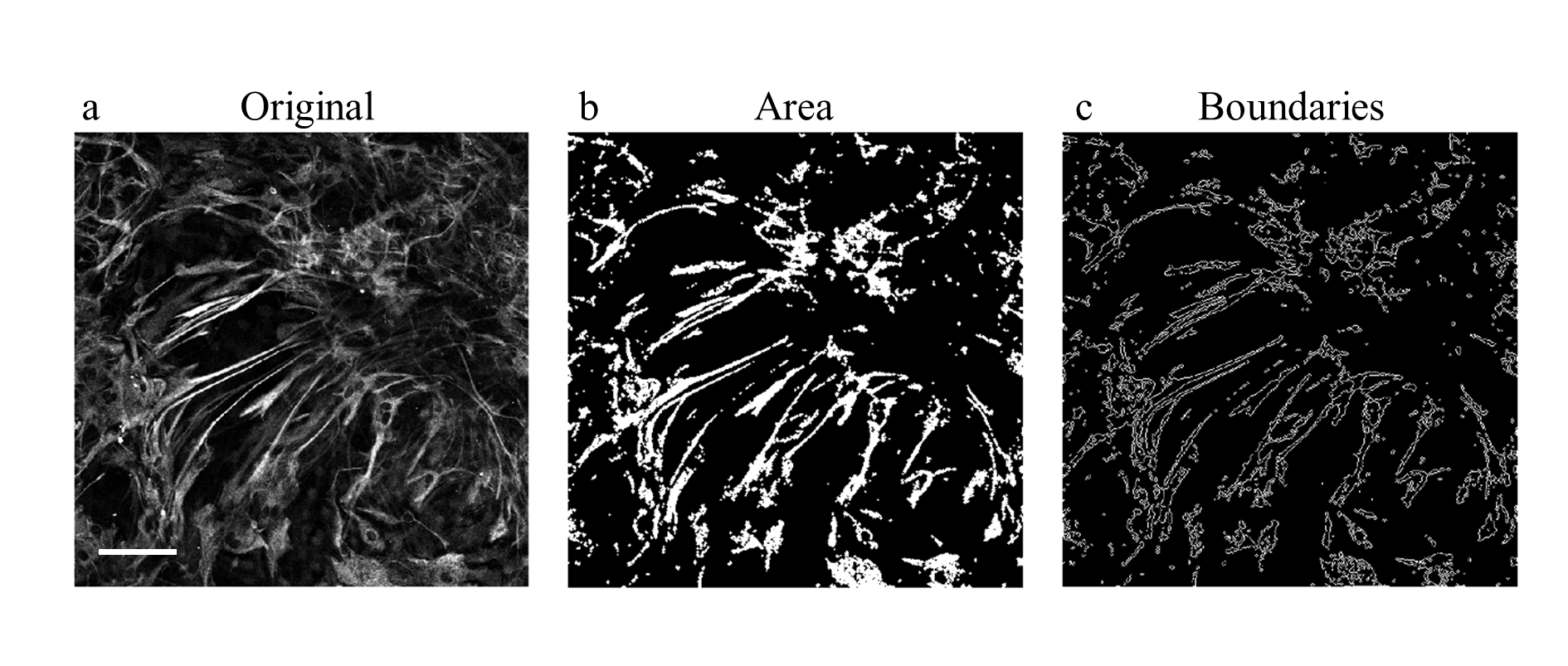

Supplement: S2 Fig — A representative image of a fixed neuron-astrocyte co-culture through the image processing steps for morphological analysis score calculation. (a) The original GFAP staining image. (b) The area of cell bodies. (c) Cells boundaries. Scale bar 100 μm. Areas and boundaries were determined by applying the algorithm described in the Materials and Methods section titled “Morphology analysis”. The morphology score of every image (control and test) was calculated as the ratio of the power (sum over all white pixels) of the boundary to the power of the total cell area. (TIF) [file pone.0203761.s002.tif]

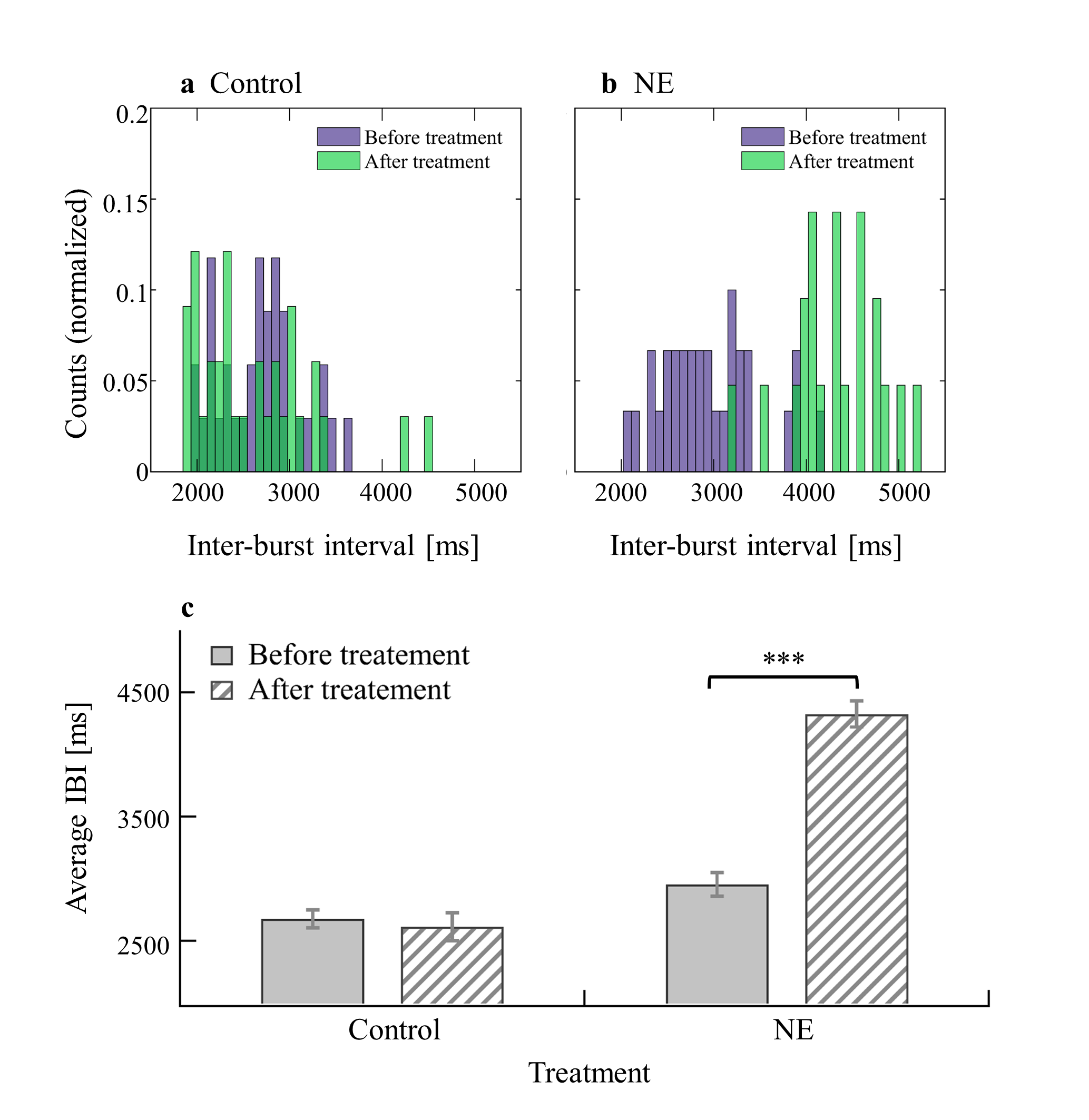

Supplement: S3 Fig — (a) IBI distribution of neurons in control experiment. (b) IBI distribution of neurons in the presence of NE. Bin width for all datasets is 87 ms. (c) Average IBI of control and NE samples before and after treatment. Error bars represent SEM. Statistical significance of differences between IBI distributions were measured using two-tailed MWU test; *** indicates p<0.001. For analysis of calcium imaging data, the fluorescence traces of all identified neurons in the field of view were averaged in order to measure neuronal network activity relying on the highly synchronized character of neuron traces. Bursts and IBIs were measured by applying Hill-Valley analysis on the averaged neuronal trace. (TIF) [file pone.0203761.s003.tif]

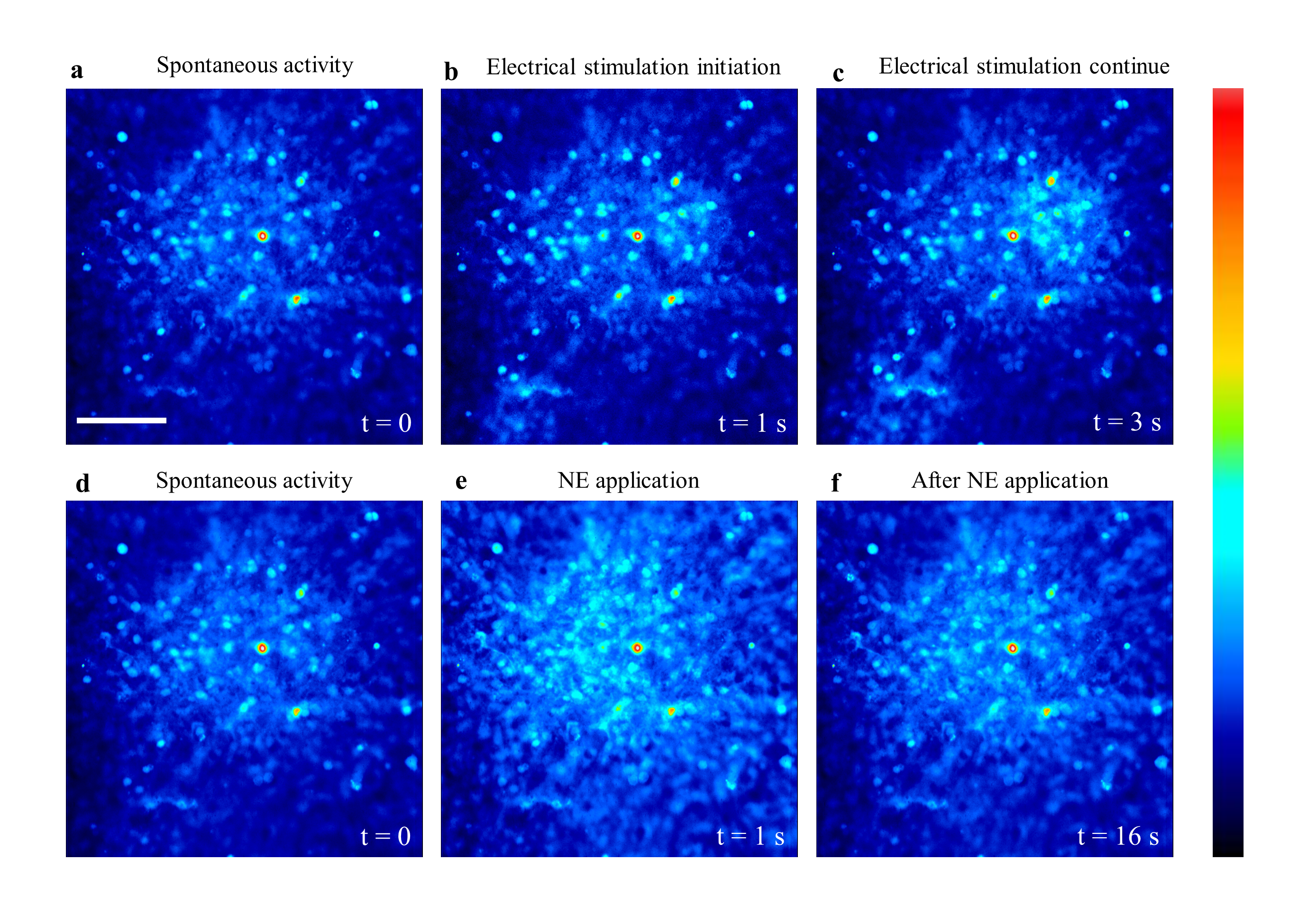

Supplement: S4 Fig — Temporally ordered selected frames from a movie of calcium imaging recorded under the influence of (a-c) ES and (d-f) NE. (a) Spontaneous activity before the application of ES. The fluorescence is equally spread across the center of the frame. (b) Initiation of ES application on the culture. Electrical current was applied at two microelectrodes (top right and lower left). Stimulation parameters: 2 stimulating electrodes, 25 μA per electrode, 10 Hz. The fluorescence shows two sources of calcium activity, aligned to the stimulating electrode locations. (c) Spreading of the ES onto the network. Fluorescence centers grow wider. (d) Spontaneous activity before the application of NE. The fluorescence is spread across the center of the frame. (e) Calcium image at the time of NE application. The fluorescence intensity grows simultaneously through the whole area, including the margins that did not show fluorescence before. (f) At 10 s after the application of NE. The increased fluorescence is starting to decay yet is still higher than the spontaneous activity baseline. These images were collected from the same recording and at the same times as the calcium traces shown in Fig 2A and 2G. The traces extracted from this recording can be seen in Fig 2A and 2G. Scale bar 100 μm. Culture age 16 DIV. (TIF) [file pone.0203761.s004.tif]

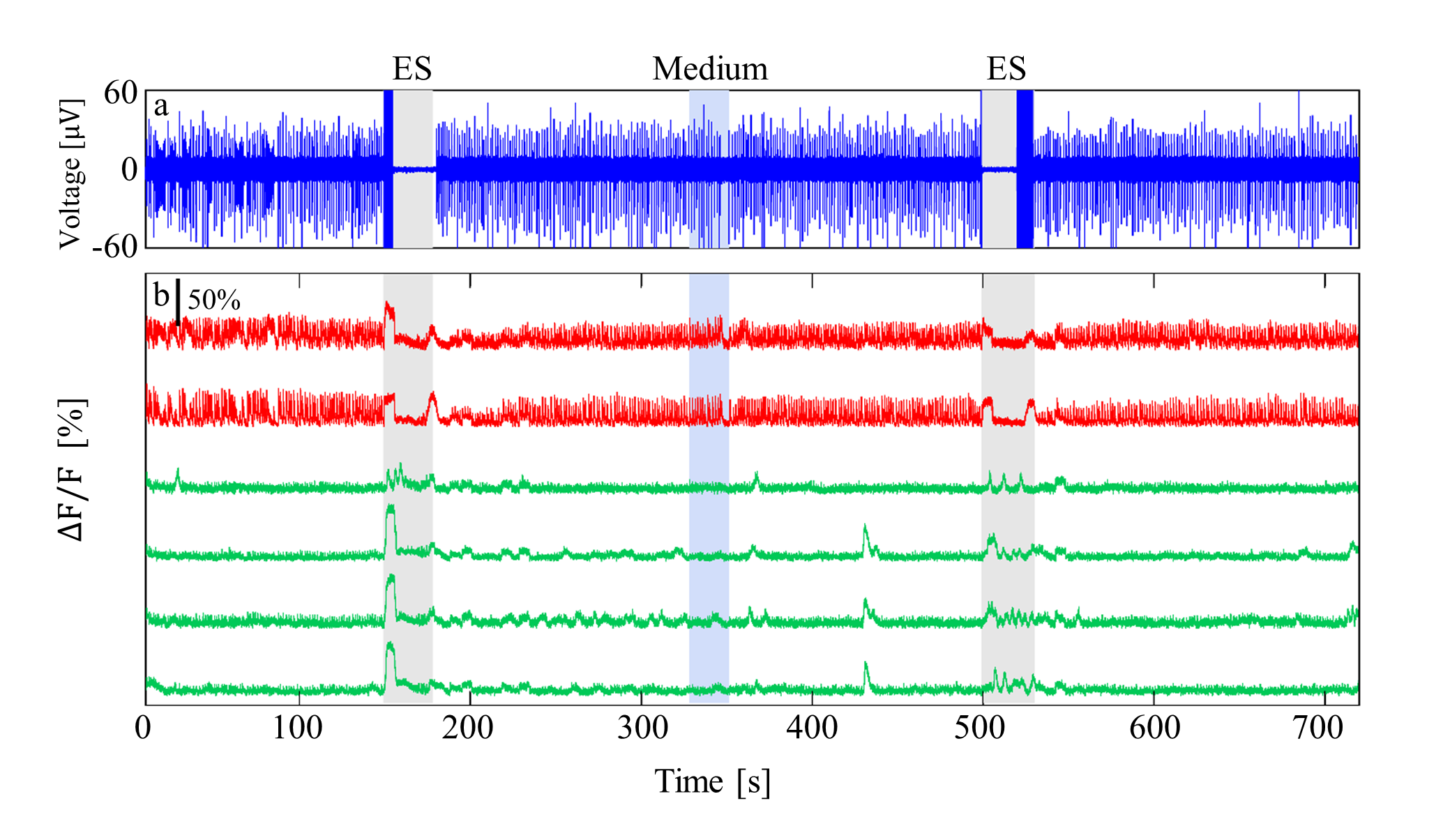

Supplement: S5 Fig — (a) Extracellular neuronal voltage recording with MEA from a representative electrode. (b) Ca2+ traces of selected neurons (red) and astrocytes (green). Periods of ES are marked by gray rectangles. Stimulation parameters: 2 stimulating electrodes, 25 μA/electrode, 10 Hz, 30 s. During the ES, the voltage recording is perturbed. Time of fresh medium addition is marked by the blue rectangle. Culture age 16 DIV. (TIF) [file pone.0203761.s005.tif]
